# Molecular Basis for Recognition of Dilysine Trafficking Motifs by COPI

**DOI:** 10.1016/j.devcel.2012.10.017

**Published:** 2012-12-11

**Authors:** Lauren P. Jackson, Michael Lewis, Helen M. Kent, Melissa A. Edeling, Philip R. Evans, Rainer Duden, David J. Owen

**Affiliations:** 1 Cambridge Institute for Medical Research, Department of Clinical Biochemistry, University of Cambridge, Hills Road, Cambridge CB2 0XY, UK; 2 Medical Research Council Laboratory of Molecular Biology, Hills Road, Cambridge CB2 0QH, UK; 3 Institute of Biology, Center for Structural and Cell Biology in Medicine, University of Lübeck, Ratzeburger Allee 160, 23562 Lübeck, Germany

## Abstract

COPI mediates retrograde trafficking from the Golgi to the endoplasmic reticulum (ER) and within the Golgi stack, sorting transmembrane proteins bearing C-terminal KKxx or KxKxx motifs. The structure of KxKxx motifs bound to the N-terminal WD-repeat domain of β'-COP identifies electrostatic contacts between the motif and complementary patches at the center of the β'-COP propeller. An absolute requirement of a two-residue spacing between the terminal carboxylate group and first lysine residue results from interactions of carbonyl groups in the motif backbone with basic side chains of β'-COP. Similar interactions are proposed to mediate binding of KKxx motifs by the homologous α-COP domain. Mutation of key interacting residues in either domain or in their cognate motifs abolishes in vitro binding and results in mistrafficking of dilysine-containing cargo in yeast without compromising cell viability. Flexibility between β'-COP WD-repeat domains and the location of cargo binding have implications for COPI coat assembly.

## Highlights

► Dilysine motifs bind the top surface of β'- and α-COP N-terminal WD-repeat domains ► Mutation of key binding site residues abolishes in vitro binding to dilysine motifs ► Loss of binding site prevents retrograde trafficking of dilysine motifs in vivo ► Mutants lacking dilysine motif binding site can transport other retrograde cargoes

## Teaser

COPI facilitates trafficking of transmembrane proteins bearing C-terminal KKxx or KxKxx motifs between the Golgi and the ER and within the Golgi stacks. Jackson et al. now provide molecular characterization of COPI cargo binding through elucidation of the structure of β'-COP N-terminal WD-repeat domain in complex with a KxKxx motif.

## Introduction

In addition to its essential role in membrane trafficking (Duden, 2003), COPI has been implicated in lipid homeostasis (Beller et al., 2008), viral replication (Cureton et al., 2012), and pathogen entry (Guo et al., 2008; Misselwitz et al., 2011). COPI facilitates retrieval of endoplasmic reticulum (ER) resident proteins from the Golgi to the ER, cycling of proteins between the ER and Golgi, and retrograde transport of proteins within the Golgi stack (Popoff et al., 2011). Many type I transmembrane proteins transported by COPI bear C-terminal dilysine-based motifs (Jackson et al., 1990; Letourneur et al., 1994; Townsley and Pelham, 1994). Lysines are strictly required at the -3 and -4 (KKxx) or -3 and -5 (KxKxx) positions relative to the C terminus and cannot be replaced by histidines or arginines (Jackson et al., 1990; Schröder-Köhne et al., 1998). Transplanting KKxx (Nilsson et al., 1989) or KxKxx motifs (Jackson et al., 1990) onto CD4/CD8 reporters results in ER retention, whereas removal of dilysine motifs from ER resident proteins results in cell surface expression (Pääbo et al., 1986). Proteins with dilysine motifs include yeast Wbp1 (Gaynor et al., 1994) and mammalian OST48 (Silberstein et al., 1992), which are essential components of *N*-linked oligosaccharyl tranferase complexes in the ER, and yeast Emp47 (Schröder et al., 1995) and its mammalian ortholog, ERGIC-53, which cycle lumenal glycoproteins between the ER and Golgi (Schröder et al., 1995; Teasdale and Jackson, 1996; Schindler et al., 1993). Certain viral glycoproteins also contain dilysine motifs (Pääbo et al., 1986; Goepfert et al., 1997), allowing viruses to circumvent host immune defenses (Pääbo et al., 1987).

The heptameric COPI complex (α/β/β'/γ/δ/ε/ζ) is recruited en bloc onto Golgi membranes (Hara-Kuge et al., 1994) but can be divided conceptually into two subcomplexes. The β/δ/γ/ζ F-subcomplex is structurally and functionally similar to AP clathrin adaptor complexes (Yu et al., 2012). The α/β'/ε B-subcomplex has been likened to clathrin because α-COP, β'-COP, and clathrin contain N-terminal WD-repeat (also referred to as β-propeller) domains and C-terminal α-solenoid domains and because recent studies have suggested that β'-COP, like clathrin, can form trimers (Lee and Goldberg, 2010). Whereas β/δ/γ/ζ and the AP complexes evolved from a common ancestor (Schledzewski et al., 1999), α- and β'-COP evolved independently of clathrin (Schledzewski et al., 1999). Very little is known about the molecular mechanisms of cargo recognition in the COPI-mediated retrograde pathway as compared to post-Golgi clathrin-mediated pathways. Controversy has long existed in the literature regarding the basis of dilysine cargo binding. Initial studies indicated that dilysine motifs bind the F-subcomplex (Harter et al., 1996). Other reports implicated components of the B-subcomplex (Cosson and Letourneur, 1994; Lowe and Kreis, 1995; Fiedler et al., 1996; Schröder-Köhne et al., 1998; Eugster et al., 2004; Béthune et al., 2006). Subsequent yeast genetic and two hybrid evidence led to the proposal that N-terminal WD-repeat domains of α- and β'-COP were responsible for differential binding to KKxx and KxKxx motifs, respectively (Eugster et al., 2004; Schröder-Köhne et al., 1998). Defects in KKxx cargo trafficking were observed in temperature sensitive yeast strains harboring mutations that cause protein misfolding (data not shown) in the α-COP N-terminal WD-repeat domain (Letourneur et al., 1994; Schröder-Köhne et al., 1998) or in strains lacking the domain altogether (Eugster et al., 2000). Similarly, deletion of the β'-COP N-terminal WD-repeat domain resulted in missorting of KxKxx signals, and loss of both α- and β'-COP domains is lethal in yeast (Eugster et al., 2004). However, a recent X-ray crystal structure of an αβ'-COP subcomplex instead proposed that the β'-COP N-terminal WD-repeat domain undertakes a structural role by forming a trimer that constitutes the vertex of a COPI cage (Lee and Goldberg, 2010).

We demonstrate that N-terminal WD-repeat domains of α- and β'-COP directly bind dilysine motifs and establish the molecular bases for the interactions using X-ray crystallography, structure-directed mutagenesis, and isothermal titration calorimetry (ITC). Finally, we show that dilysine cargo trafficking is mediated through binding of the motifs to the N-terminal WD-repeat domains of α- and β'-COP in vivo.

## Results

### Structure of β'-COP 1-304 with a KxKxx Motif

ITC experiments conclusively showed that dilysine motifs bound robustly with a low micromolar K_D_ to the β'-COP N-terminal WD-repeat domain (see next section). Attempts to crystallize this domain were made in the presence of a variety of KKxx- and KxKxx-containing peptides based on endogenous motifs (Table S1 available online). The first structure obtained was that of yeast β'1-304His6 in the presence of the CTFKKTN motif derived from yeast Wbp1 (Table 1; Supplemental Experimental Procedures; Figures S1D–S1G). This structure, solved at 1.8 Å resolution by SIRAS, showed that the WD-repeat domains pack one on top of the other in the lattice. The C-terminal His6 tag from one molecule reaches to the top surface of its neighbor, specifically occupying a charged surface patch (Figure S1G). This structure suggested a potential binding site for short basic linear motifs, particularly highlighting the importance of a terminal carboxylate group and backbone carbonyl binding. The CTFKKTN peptide included in crystal trials was not visible in the density but was required for crystallization, likely forming weak interactions that facilitated crystal packing. As a result, we used an untagged β'1-304 construct in all further studies and subsequently obtained structures of β'1-304 in complex with CTFKTKTN and CTFKKTN peptides. Because both structures reveal the same mode of binding via the carboxy terminii and -3 lysines of motifs, we present here the structure of β'1-304 with its preferred ligand, CTFKTKTN.

**Figure 1:**
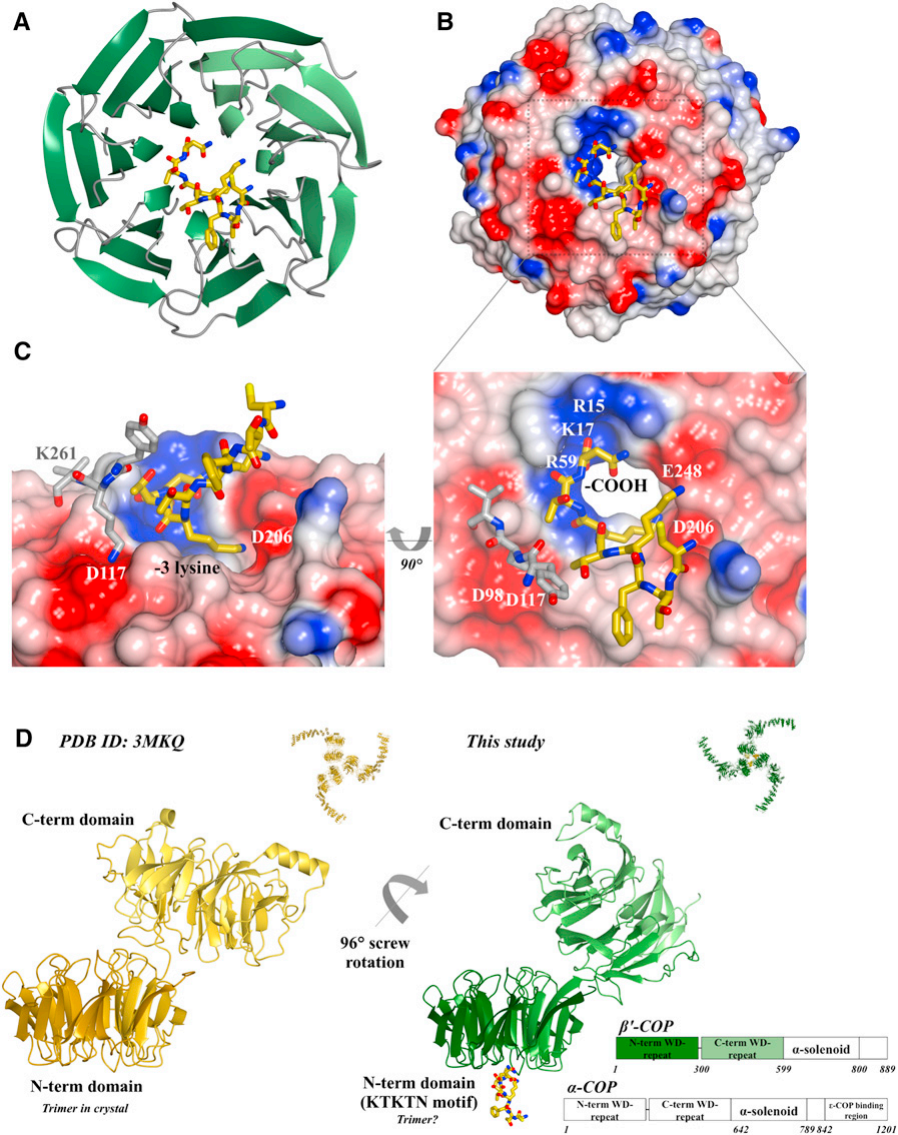
Structures of β'-COP 1-304 and 1-604 with a KxKxx Motif (A and B) Seven-bladed WD-repeat domain of yeast β'1-304 (green) with KTKTN peptide (yellow) in ribbon diagram (A) and electrostatic surface potential (B) views, contoured from -0.5V (red) to +0.5V (blue). (B, inset) The peptide carboxy terminus sits in a groove formed by β'-COP R15, K17, and R59, whereas the -3 lysine interacts with D206/E248. (C) K261 from a symmetry copy (gray residues) occupies the negative patch formed by D98/D117 (view rotated 90°). (D) The linker between β'-COP N- and C-terminal domains is flexible. β'1-604 (PDB ID code 3MKQ) is shown from gold to lemon (N to C terminus); β'1-604 with CTFKTKTN in this study is shown from dark to light green (N to C terminus) with peptide in yellow. See also Figure S2 and Tables S1 and S2.

All eight residues of the peptide are visible in the 1.8 Å resolution complex (Figures 1A–1C and S1A–S1C; Table 1). Interaction between β'1-304 and the KxKxx motif is mediated primarily by electrostatic contacts. The peptide carboxy terminus is located on a positively charged patch formed by guanidinium groups of R15 and R59 and the amino group of K17 in the center of the top surface of the WD-repeat domain. The -3 lysine interacts with a negatively charged patch formed from carboxylate groups of D206 and E248. In a negative patch formed by D98 and D117, a lysine residue (K261) from a symmetry copy of the domain (Figure 1C) has displaced the -5 lysine found in the peptide motif. This is one of two important crystal contacts, and attempts to crystallize K261 mutants with CTFKTKTN failed. The second critical crystal contact is a disulfide bond formed by oxidation between the peptide cysteine and C220 in a symmetry copy of β'1-304. Attempts to grow crystals in the presence of a reducing agent failed, highlighting its necessity in crystallization. The final key structural feature is the location of the -2, -3, and -4 carbonyl groups of the motif, which project down into a basic groove.

### The WD-Repeat Domains of β'-COP Exhibit Inherent Flexibility

A recently published crystal structure of β'-COP (Protein Data Bank [PDB] ID code: 3MKQ) suggested that its N-terminal WD-repeat domains mediate formation of a trimer (Lee and Goldberg, 2010). In contrast, we do not observe trimer formation of the N-terminal WD-repeat domain in any of our structures of either β'1-304 with different peptides (Supplemental Experimental Procedures) or β'1-604 with CTFKTKTN (Figures 1D and S2; Table 1). As in published work (Lee and Goldberg, 2010), we find no evidence of trimer formation in solution by multiangle light scattering (Supplemental Experimental Procedures), suggesting an approximate millimolar K_D_. However, our β'1-604 structure demonstrates that there must be significant conformational flexibility between the two WD-repeat domains of β'-COP because the C-terminal domain undergoes a ∼96° screw rotation (Figures 1D and S2) relative to 3MKQ, when the structures are superposed on their N-terminal domains. If the interface between the C-terminal WD-repeat domain and α-solenoid is rigid, rotation of the C-terminal domain by ∼96° would rotate each α-solenoid domain by ∼180° relative to 3MKQ, resulting in a trimer of the opposite hand (Figures 1D and S2).

### β'- and α-COP N-Terminal WD-Repeat Domains Bind Dilysine Motifs In Vitro

**Figure 2:**
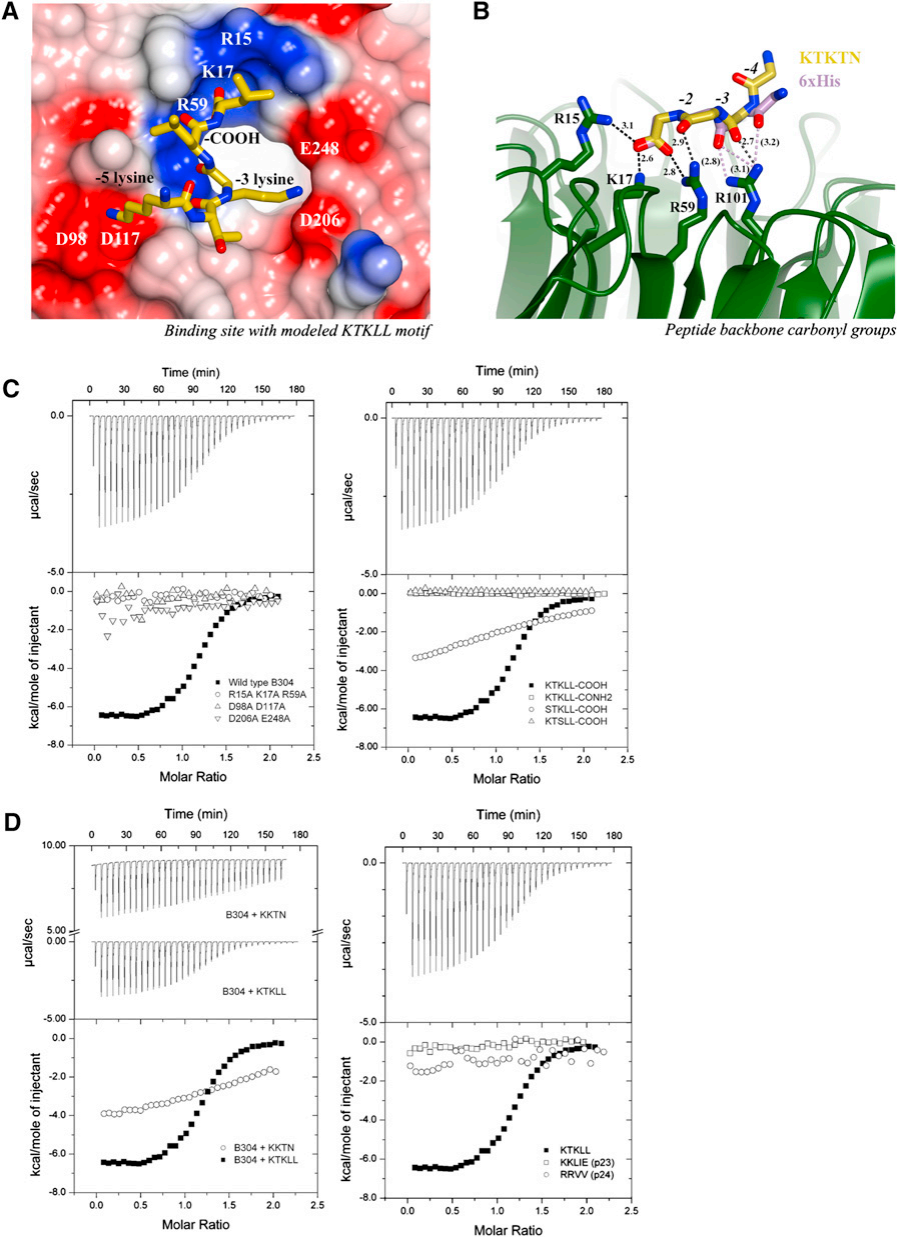
Key Residues in β'-COP and the Dilysine Motif Are Required for In Vitro Binding (A) β'-COP with modeled yeast Emp47 KTKLL motif. (B) The motif carboxy terminus and -2 and -3 carbonyl groups project down to the basic patch of R15/K17/R59/R101. The CTFKTKTN backbone is shown in yellow with hydrogen bonds and distances in dark gray; His6 backbone from β'1-304His6 structure (Supplemental Experimental Procedures) is shown in purple with hydrogen bonds in dotted purple and distances in parentheses. (C) Wild-type yeast β'-COP 1-304 binds KTKLL with a K_D_ of 6.8 ± 2.6 μM; structure-based point mutants exhibited no measurable binding (K_D_ < 300 μM). Loss of motif carboxy terminus or -3 lysine abolishes measurable binding to β'-COP and loss of the -5 lysine resulted in substantially weaker binding (K_D_ ∼160 μM). (D) Affinity of β'1-304 for the KKTN motif is approximately an order of magnitude lower (K_D_ ∼85 μM) than for the KTKLL motif. β'1-304 exhibits no measurable binding to the KKLIE motif from p23 or the RRVV motif from p24 proteins. See also Figure S3.

Having proposed residues as important for binding of the β'-COP N-terminal WD-repeat domain to KxKxx motifs (Figures 1C, 2A, and 2B), we tested our model in solution using a combination of ITC and structure-based mutagenesis. For all biophysical experiments, the KxKxx motif from wild-type yeast Emp47 (RQEIIKTKLL) was used instead of the synthetic peptide used in crystallization, demonstrating the disulfide bond observed in the structure is not required for binding in solution. Wild-type yeast β'1-304 binds the KTKLL motif with a K_D_ of 6.8 ± 2.6 μM (Figure 2C). β'-COP exhibits differential binding to KKxx and KxKxx signals in vitro: the affinity for the Wbp1 KKTN motif (Figure 2D) is weaker by an order of magnitude (K_D_ ∼85 μM), and hence we hypothesize that a lysine at the -4 position likely cannot interact optimally with the patch formed of D98 and D117 (Figure 2A). For two reasons, we cannot draw conclusions regarding preference of β'-COP for a -5 lysine over a -4 lysine from comparing structures of β'-COP 1-304 with different peptides. First, formation of the disulfide bond between the peptide cysteine and C220 “pulls” the N-terminal end of the peptide toward a symmetry copy in the crystal lattice, altering the backbone conformation. Second, K261 from a symmetry copy occupies the patch formed of D98 and D117 in both structures. These features cause lysines at either the -4 or -5 positions to relocate into the solvent channel, where they are not visible in unbiased electron density (Figure S1B). However, the importance of the D98/D117 patch for -5 lysine binding is confirmed by mutagenesis (below).

We next constructed mutants to confirm importance of specific residues in the motif-binding site. The β'-COP R15A K17A R59A mutant was designed to disrupt binding to the carboxy terminus of the motif, and the D206A E248A mutant was intended to disrupt binding to the -3 lysine. Based on indirect evidence from structures, we predicted a D98A D117A mutant would abolish interaction with the -5 lysine. This prediction was based on the position of a histidine residue equivalent to the -5 lysine in the β'1-304His6 structure (Figure S1G) and of the symmetry copy K261 in the β'1-304 structure (Figure 1C), both of which occupy the D98/D117 patch. Binding of all three mutants to RQEIIKTKLL was undetectable by ITC (K_D_ < 300 μM) (Figure 2C), without affecting overall protein fold as judged by circular dichroism (CD) and gel filtration elution profiles (data not shown).

We also tested the effect of altering key determinants in the KxKxx motif (Figure 2C). Wild-type β'1-304 exhibits no measurable binding to an amidated peptide (KTKLL-CONH_2_) or to KTSLL and weak binding to STKLL (K_D_ ∼160 μM). Together with our structures, these data indicate the key motif determinants are the -3 lysine and carboxy terminus, which are present in both KxKxx and KKxx motifs. In addition to the carboxylate group binding to the basic patch formed of R15/K17/R59, two carbonyl groups from the -2 and -3 positions in the motif backbone must fit into a groove formed by R59 and R101; the carbonyl group at the -4 position also likely contacts R101, as observed in the β'1-304His6 structure (Figure 2B). Fulfilling these two requirements restricts the placement of a lysine residue such that it can only be accommodated in the patch formed by D206 and E248 when located two residues away from the carboxy terminus (i.e., at the -3 position). Testing of potential variant basic motifs further confirmed these results: the KKLIE peptide from p23, which lacks a -3 lysine, does not bind β'-COP (Figure 2D). In agreement with previous work (Fiedler et al., 1996), arginines cannot replace lysines: we could detect no binding to an RRVV peptide from p24 (Figure 2D). When carbonyl groups in the peptide backbone sit in the basic groove, an arginine side chain is too long to be accommodated at the -3 position.

**Figure 3:**
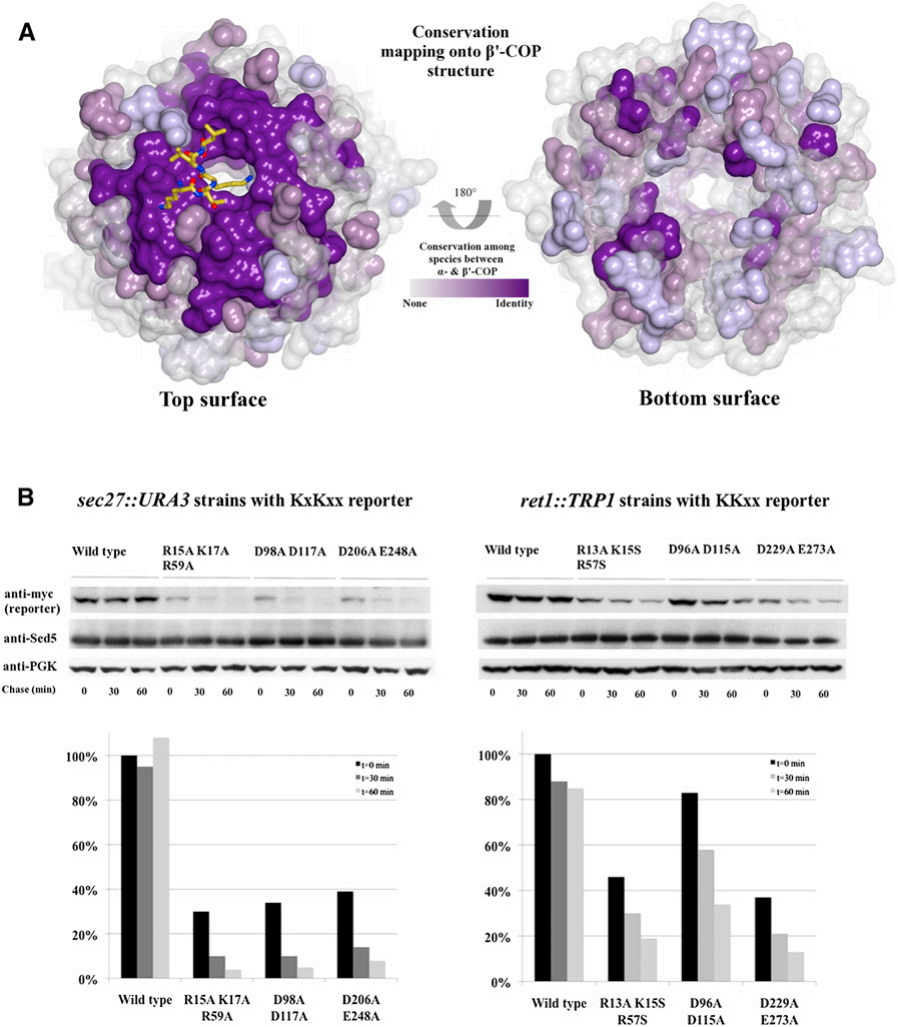
Loss of the Motif Binding Site in Either α- or β'-COP Alters Trafficking of a Dilysine Reporter in Yeast (A) The dilysine binding pocket is conserved between α- and β'-COP from yeast to humans. Top-down view of the N-terminal WD-repeat domain (peptide in yellow), colored by residue conservation from transparent gray (no conservation) to dark purple (absolute identity). (B) Wild-type-replaced *SEC27::URA3* cells maintained KxKxx reporter levels, whereas all point-mutant-replaced strains exhibited lower steady-state levels and increased vacuolar degradation of the reporter over time. Blots and bar graphs indicate a representative experiment with percentages reported relative to wild-type levels prior to the chase (t = 0 min). Analogous mutations in the *RET1* binding site hindered trafficking of a KKxx reporter construct. See also Figure S4.

α- and β'-COP arose from a single gene by duplication and thus possess conserved domain architecture. The N-terminal WD-repeat domains exhibit 51% similarity and 18% identity at the amino acid level (data not shown), and hence we should be able to reliably model the α-COP structure. Because all of the residues in the β'-COP dilysine motif binding site are conserved in α-COP (Figure 3A), the α-COP domain must possess analogous charged patches on its top surface (Figure S3C), pointing to a conserved mechanism of motif binding. Using structures of β'-COP/KxKxx motif complexes, we generated a homology model of residues 1–327 of yeast α-COP and modeled the interaction with the Wbp1 KKTN motif (Figure S3). The three common motif determinants (carboxy terminus, backbone carbonyl groups, and -3 lysine) should bind in the same way, with the only difference being the preference for a lysine at the -4 position.

As predicted, analogous mutations to those proposed for β'-COP introduced into yeast α-COP gave comparable results for KKTN motif binding as determined by ITC, without affecting protein fold (CD and gel filtration profiles; data not shown). R15A K17S R59S disrupted binding to the motif carboxy terminus, and D229A E273A disrupted binding to the -3 lysine (Figure S3B). Although we have no experimental evidence for α-COP and indirect evidence from β'-COP structures, we attempted to model how the -4 lysine binds on the α-COP surface. By analogy with β'-COP, we hypothesized that D96 and D115 comprise the binding site for the -4 lysine and designed a D96A D115A mutant. These two residues are absolutely conserved between both α- and β'-COP from yeast to humans (Figure 3A). In addition, the short motif would be able physically to bridge the distance between D96/D115 and the other two patches known to be important for binding. The closest additional negative patch on the surface of our model (D296/D315) is located too far away (∼13Å) from the C-α of the -3 lysine to be involved. As predicted, the D96A D115A mutant abolished binding to KKxx motifs in vitro (Figure S3B).

### α- and β'-COP Traffic Dilysine-Based Reporter Constructs in Yeast

Finally, we tested our structural model of dilysine motif binding by COPI in *Saccharomyces cerevisiae*. Using homologous recombination, we generated strains by replacing endogeneous *SEC27* (β'-COP) or *RET1* (α-COP) genes with structure-based point mutants described above and then introduced dilysine reporter constructs. We then observed how wild-type-replaced and point-mutant-replaced strains transported dilysine cargo. Based on published information (Gaynor et al., 1994; Letourneur et al., 1994; Schröder-Köhne et al., 1998), we predicted wild-type-replaced strains would recycle reporters back to the ER, whereas mutant strains would fail to recycle reporters, leading to vacuolar degradation. Results indicated that our structural model correctly describes interaction of the β'-COP N-terminal WD-repeat domain with dilysine motifs. All mutations in *SEC27* that disrupt binding to the KxKxx motif resulted in significantly lower steady-state levels of the KxKxx reporter. Reporter levels in all mutant strains are 30%–40% of wild-type levels prior to the cycloheximide chase (Figure 3B). One hour after the chase, the majority of the reporter (∼90%–95%) has been degraded in the vacuole. In contrast, the wild-type-replaced strain maintained similar levels of the reporter over the time course of the experiment. A KKxx reporter (Figure S4A) also exhibited lower steady-state levels and degradation in the vacuole. These phenotypes agree with structural and in vitro binding results, confirming that β'-COP can bind both types of motif (Figure 2D).

Likewise, our α-COP homology model correctly predicted critical residues for binding the carboxy terminus (R13A K15S R57S mutant) and -3 lysine (D229A E273A mutant) of KKxx motifs. Disrupting these patches in *RET1* resulted in steady-state KKxx reporter levels of ∼40% and loss of ∼80% of the reporter over time, compared to wild-type levels (Figure 3B). The effect is significant but less pronounced than effects on degradation of the KxKxx reporter in *sec27::URA3* mutant strains. Both in vitro and in vivo data suggest that β'-COP can bind to and thus transport KKxx motif-containing cargo (endogenous and reporter-based) in the absence of a functional α-COP motif binding site. Because the KKxx motif is more prevalent in yeast, our results together suggest that β'-COP carries the bulk of reporter in our strains.

We observed a less significant steady-state effect (∼85% of wild-type levels) and less degradation (loss of ∼65%) in the *ret1* D96A D115A mutant predicted to bind the -4 lysine, suggesting this mutant still shows functionally significant binding to KKxx motifs. This reflects uncertainty inherent in the α-COP homology model. We do not know the absolute position and orientation of the α-COP determinant residues located on the conserved patch, and other contacts may be involved in -4 lysine binding. Indeed, the charge and shape of the D229/E273 patch may be able to accommodate both the -3 and -4 lysines. An X-ray structure of the α-COP N-terminal WD-repeat domain with KKxx motif will be required to understand the -4 lysine binding preference. Thus far, the α-COP N-terminal domain from multiple organisms (yeast, fly, and humans) has proven refractory to crystallization despite extensive efforts (Supplemental Experimental Procedures).

One possible explanation for the observed phenotypes would be that our point mutations have disrupted formation and thus all functions of COPI. However, in addition to confirming that point mutations did not disrupt folding of recombinant N-terminal WD-repeat domains, three lines of evidence suggested that the COPI coat was intact and functional in mutant strains (Figure S4). First, COPI subunits (F-/B-subcomplexes) were expressed at similar levels in mutant and wild-type replacement strains (Figure S4C), demonstrating that point mutations have not disrupted complex formation. Second, none of the mutant strains exhibited temperature sensitivity up to 37°C (Figure S4D), even though both *SEC27* and *RET1* are essential genes. Finally, in point-mutant-replaced strains, the syntaxin Sed5, which cycles between the ER and Golgi (Wooding and Pelham, 1998; Weinberger et al., 2005), is present at levels found in wild-type cells (Figures 3B, S4A, and S4B), indicating that COPI is functional for non-dilysine-based retrograde transport from the Golgi.

Finally, we generated a yeast strain in which both the α- and β'-COP N-terminal WD-repeat domains had lost the ability to bind the carboxy terminus of dilysine motifs (*sec27::URA3* R15A K17A R59A *ret1::TRP1* R13A K15S R57S). Although viable at 37°C, the ability of this mutant to traffic both KKxx and KxKxx reporter constructs was severely impaired (Figure S4E). The inability to support retrograde dilysine-based transport causes a slight growth defect at 37°C, resulting in smaller colonies as compared to wild-type, but this mutant is not lethal.

## Discussion

A molecular explanation of cargo binding by the COPI coat complex has long proven elusive. We have demonstrated here how the N-terminal WD-repeat domains of COPI B-subcomplexes recognize the most commonly used retrograde trafficking signals, dilysine-based motifs. Unlike previously used temperature-sensitive and deletion strains that result in general protein misfolding, our highly specific tools selectively abolish a single function of coatomer and will open the way for in vivo investigation of retrograde trafficking, including roles and importance of dilysine-based cargo in COPI coat recruitment and other cellular functions.

COPI coat assembly requires both membrane and cargo binding (Hara-Kuge et al., 1994; Lowe and Kreis, 1995; Bremser et al., 1999). To bind their cargo, α- and β'-COP N-terminal WD-repeat domains must approach very close to the membrane (∼20 Å) because the minimum spacing between a transmembrane domain and C-terminal dilysine motif found in endogenous cargo is five amino acid residues. In addition to binding cargo, the β'-COP N-terminal WD-repeat domain has been proposed to form a trimer (Lee and Goldberg, 2010), and cryoelectron tomography data from reconstituted COPI-coated vesicles demonstrate that a portion of density with 3-fold local symmetry is located proximal to the membrane (Faini et al., 2012). It is tempting to speculate that this density corresponds to trimers of β'-COP (and possibly α-COP) N-terminal WD-repeat domains. Indeed, a model of KxKxx motif binding to trimeric β'-COP, the mechanism of which we have described here, indicates there is sufficient space within the proposed interface for each WD-repeat domain to bind a single KxKxx motif (Figure S2B). It is therefore possible that β'-COP N-terminal WD-repeat domains simultaneously bind cargo and mediate trimerization. However, fitting of the whole 3MKQ trimeric structure (Lee and Goldberg, 2010) or a trimer based on our structure of β'1-604 with CTFKTKTN (Figure 1D; data not shown) into tomographic electron density is poor (Faini et al., 2012). One explanation for the poor fit is the significant conformational flexibility within β'-COP as indicated by comparing our structure of β'1-604 with CTFKTKTN with the unliganded structure (Lee and Goldberg, 2010). Flexibility in the COPI coat is in line with cryo-EM reconstructions (Yip and Walz, 2011) and cryoelectron tomography data (Faini et al., 2012). It may be that once the N-terminal WD-repeat domains are anchored to the membrane by cargo binding, flexibility between the two WD-repeat domains of β'-COP, and likely also α-COP, may facilitate assembly of polymeric coats having a range of geometries with local but no global point group symmetry. Docking of available crystal structures into tomographic maps should be aided in future by higher resolution maps, and our identification of the dilysine motif binding site should allow specific labeling of the α- and β'-COP WD-repeat domains with modified peptides so that domains in tomographic electron density can be assigned unambiguously.

Assuming that β'-COP, and by analogy α-COP, N-terminal WD-repeat domains form trimers under certain conditions and, taking published data into account, a scenario could arise in which the COPI heptamer is initially recruited onto Golgi membranes through binding of monomeric β/δ/γ/ζ F-subcomplex to Arf1-GTP (Yu et al., 2012). Any multimerization of α/β'/ε subcomplexes that could lead to coat polymerization should only be driven by extremely high local concentrations of B-subcomplexes because interactions like the very weak β'-COP trimeric interface (Lee and Goldberg, 2010) would occur only when N-terminal WD-repeat domains are highly concentrated on the membrane surface. High concentrations are possible due to avidity effects resulting from binding of B-subcomplexes both to F-subcomplexes recruited by Arf1-GTP and also directly to dilysine cargo embedded within the membrane. This model agrees with published data demonstrating how binding to both Arf1-GTP and membrane-embedded cargo is necessary for efficient COPI coat formation (Bremser et al., 1999; Faini et al., 2012; Lowe and Kreis, 1995); the cargo-containing membrane can therefore be considered as a scaffold upon which the coat forms. This model is also consistent with the observation that following coat formation, GTP hydrolysis of Arf1 results in release of Arf1 from the membrane, while polymeric COPI remains attached (Presley et al., 2002), likely because interactions of polymer-forming α- and β'-COP interfaces are maintained at high local concentrations through direct interactions with dilysine-embedded cargo. Although our yeast strain lacking binding sites on both α- and β'-COP for the carboxy terminus of dilysine motifs was viable, coat formation may be less efficient than in wild-type cells but still sufficient to support non-dilysine-based retrograde trafficking in a laboratory environment. Together with published work (Lee and Goldberg, 2010; Faini et al., 2012), our data suggest that cargo binding and coat formation by COPI may be coupled, providing a mechanism linking coat polymerization to the presence of the membrane cargo required in the retrograde pathway.

## Experimental Procedures

For constructs, antibodies, and yeast strains used in this study, see the Supplemental Experimental Procedures.

### Protein Expression and Purification

Constructs were expressed in BL21(DE3)pLysS cells (Invitrogen, Carlsbad, CA, USA) for 16–20 hr at 22°C after induction with 0.2 mM IPTG. β'-COP constructs were purified in 20 mM Tris (pH 7.4), 200 mM NaCl, and 2 mM DTT, and α-COP constructs were purified in 20 mM Tris (pH 7.4), 500 mM NaCl, and 2 mM DTT. Cells were lysed by a disruptor (Constant Systems Limited, Daventry, UK), and proteins were affinity purified using Ni-NTA agarose (QIAGEN, Hilden, Germany) or glutathione sepharose (GE Healthcare, Waukesha, WI, USA) in the relevant buffer. GST-tagged proteins were cleaved overnight with thrombin (SERVA, Heidelberg, Germany) and eluted in batch. All proteins were further purified by gel filtration on a Superdex S200 preparative column (GE Healthcare).

### Structure Determination of β'1-304 and β'1-604 with CTFKTKTN

The CTFKTKTN peptide (30 mg/ml) was mixed 1:10 with β'1-304 (final concentration 10 mg/ml) and crystallized in 0.1 M HEPES (pH 7.5), 0.5 M ammonium sulfate, and 30% v/v MPD, which was sufficient for cryoprotection. Data were collected at Diamond Light Source, beamline I04-1, on a Pilatus 2M detector and integrated using Xia2. Crystals diffracted beyond 1.8 Å resolution and were of space group P6_1_ with cell dimensions a = 129.16 Å, b = 129.16 Å, c = 60.03 Å, α = 90°, β = 90°, and γ = 120°. The structure was solved by molecular replacement using the high-resolution β'1-304His6 structure (Supplemental Experimental Procedures) as a model. Rounds of refinement and rebuilding were undertaken using phenix.refine and Coot, respectively.

β'1-604 crystallized at 10 mg/ml with CTFKTKTN (mixed 1:10 at 30 mg/ml) in 0.1M bicine (pH 9.0) and 10% MPD. Crystals were cryoprotected with 40% MPD and flash frozen by plunging in liquid nitrogen, and data were collected to 3.0 Å resolution at Diamond Light Source, beamline I04-1, using a Pilatus 2M detector. The structure was solved by molecular replacement using two successive searches: the β'1-304His6 structure to locate the N-terminal WD-repeat domain and residues 310–600 of 3MKQ to locate the C-terminal domain. Crystals were of space group P3_1_ with cell dimensions a = 127.25 Å, b = 127.25 Å, c = 59.06 Å, α = 90°, β = 90°, and γ = 120°. Crystals exhibited twinning (two domains of fractions 58.6% and 41.4%), and intensity-based twin refinement in REFMAC5 was used for initial rounds of refinement and model building. Final refinement runs and structure validation were performed in phenix.refine.

### Isothermal Titration Calorimetry

ITC experiments were conducted on a Microcal VP-ITC machine (GE Healthcare) at 10°C. Molar peptide concentration in the syringe was ten times that of protein in the cell. β'-COP constructs were gel filtered into 50 mM HEPES (pH 7.4) and 100 mM NaCl, and α-COP constructs were gel filtered into 50 mM HEPES (pH 7.4) and 300 mM NaCl prior to runs. Titration data were analyzed in ORIGIN to obtain values for stoichiometry (N), equilibrium association constant (K_a_), and enthalpy of binding. All experiments were carried out at least three times with appropriate standard deviations reported.

## Figures and Tables

**Table 1:** Data Collection, Phasing, and Refinement Statistics

	β'1-304His6 Native	β'1-304His6 Mercury Derivative	β'1-304 with KTKTN	β'1-604 with KTKTN
**Data Collection**				

Beamline	Diamond I02	Diamond I02	Diamond I04-1	Diamond I04-1
Space group	C2	C2	P6_1_	P3_1_
Wavelength (Å)	1.0	1.0	0.917	0.917

**Cell Dimensions (Å)**				

*a*, *b*, *c* (Å)	171.6, 50.5, 74.5	171.7, 50.5, 74.4	129.2, 129.2, 60.0	127.3, 127.3, 59.1
*α*, *β*, *γ* (°)	90.0, 104.0, 90.0	90.0, 104.0, 90.0	90.0, 90.0, 120.0	90.0, 90.0, 120.0
Resolution range (Å)	49.0–1.80 (1.80)	48.4–1.75 (1.75)	56.0–1.73 (1.73)	63.6–2.96 (2.96)
R_merge_	0.057 (0.101)	0.058 (0.206)	0.131 (0.662)	0.062 (0.568)
Mean *I*/*σI*	24.4 (10.2)	24.5 (7.7)	8.7 (2.7)	15.8 (2.0)
Completeness (%)	96.9 (81.6)	91.5 (61.3)	100.0 (100.0)	99.3 (99.9)

**Refinement**				

Resolution range (Å)	49.0–1.80	–	55.9–1.78	63.6–2.96
No. reflections	55,618	–	54,637	22,113
R_work_/ R_free_	0.1880/0.2233	–	0.1621/0.1799	0.2396/0.2943

**Number of Atoms**				

Protein	5,485	–	2,776	4,853
Ligand/ion	25	–	80	65
Water	403	–	231	2
Wilson B-factor (Å^2^)	14.8	–	14.1	73.1

**Rmsd from Ideal Values**				

Bond lengths (Å)	0.007	–	0.007	0.009
Bond angles (°)	1.22	–	1.21	1.38

**Ramachandran Plot**				

Favored region (%)	96.7	–	96.8	90.9
Allowed (%)	3.2	–	3.2	7.8
Outliers (%)	0.2	–	0.0	1.3
Rotamer outliers (%)	0.4	–	1.0	5.6
C-beta outliers	0	–	0	0
PDB ID code	2YNO	–	2YNN	2YNP

See also Figure S1.

## Accession Numbers

Coordinates for all structures have been deposited in the Protein Data Bank under ID codes 2YNO, 2YNN, and 2YNP.

## Supplemental Information

Document S1. Figures S1–S4, Tables S1 and S2, and Supplemental Experimental Procedures
