# Supplementary material for: Molecular Basis for Recognition of Dilysine Trafficking Motifs by COPI
Source: Dev Cell. 2012 Dec 11;23(6-12):1255–62. doi: 10.1016/j.devcel.2012.10.017 (PMC3521961; doi:10.1016/j.devcel.2012.10.017)
Supplement: Document S1. Figures S1–S4, Tables S1 and S2, and Supplemental Experimental Procedures [file mmc1.pdf]

Developmental Cell, Volume *23*

Supplemental Information

Molecular Basis for Recognition of Dilysine Trafficking Motifs by COPI

Lauren P. Jackson, Michael Lewis, Helen M. Kent, Melissa A. Edeling, Philip R. Evans, Rainer Duden, and David J. Owen

Inventory of Supplemental Materials

Figure S1, related to Table 1. Structure overview of β'1-304 with CTFKTKTN and β'1-304His6.

Figure S2, related to Figure 1. Flexibility between β'-COP WD-repeat domains facilitates formation of COPI coats having a range of geometries.

Figure S3, related to Figure 2. Model and *in vitro* data of α-COP 1-327 binding to a KKxx motif.

Figure S4, related to Figure 3. Trafficking of converse dilysine reporter constructs, COPI stability, and viability in yeast.

Table S1, related to Figure 1. Peptides used in crystallization trials with β'-COP constructs

Table S2, related to Figure 1. MALS results.

Supplementary Experimental Procedures

Supplementary References

Supplementary Materials

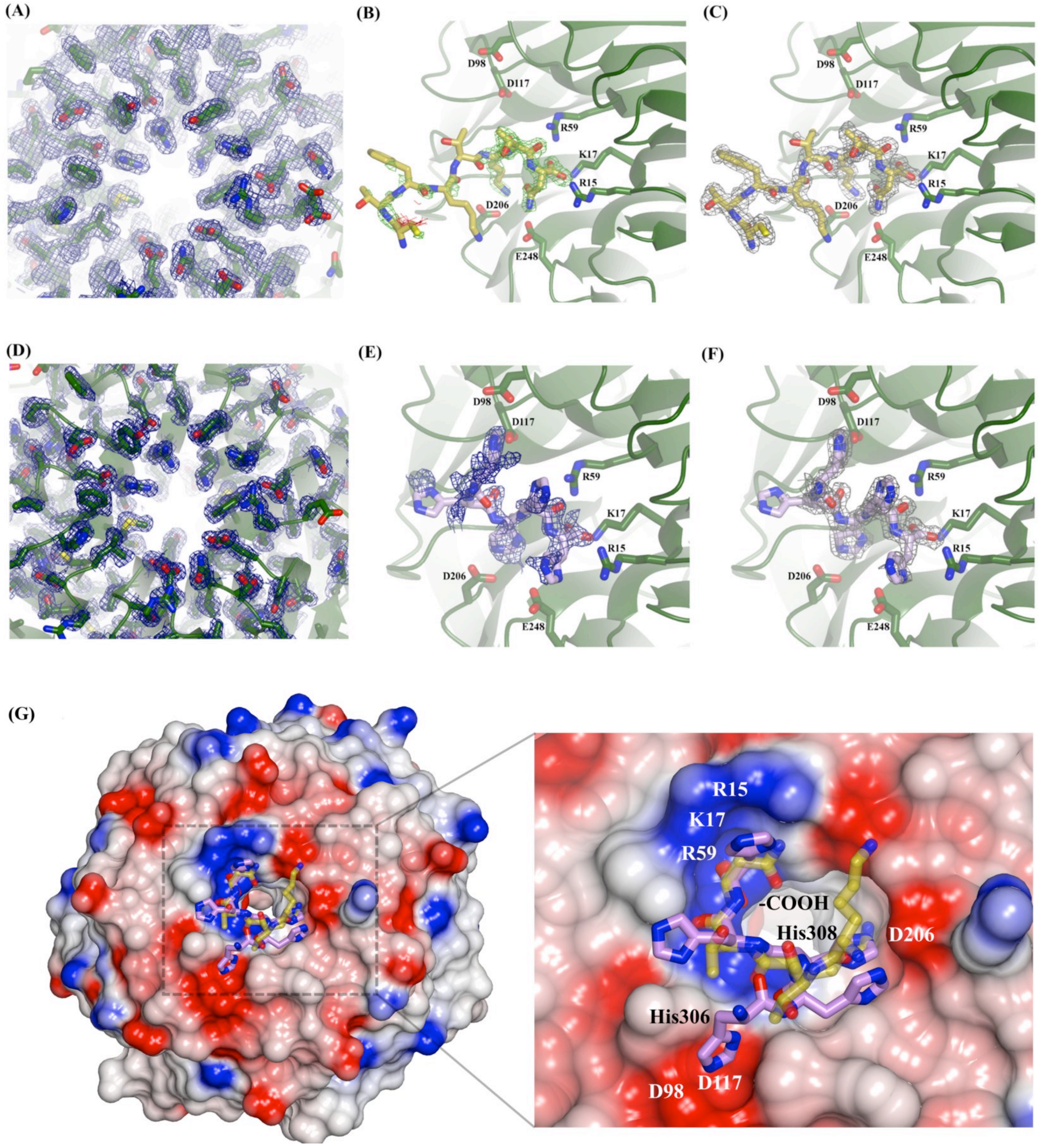

Figure S1, related to Table 1. Structure overview of β'1-304 with CTFKTKTN and β'1-304His6. Density from β'1-304 in complex with CTFKTKTN (A-C) and β'1-304His6 structure (D-F). (A) Representative $2mF_O\text{-}DF_C$ electron density contoured at 1σ is shown in blue following molecular replacement in Phaser for the WD-repeat domain. (B) $mF_O\text{-}DF_C$ density is shown for the peptide binding region following molecular replacement in Phaser. The carboxy terminus and -3 lysine are well-ordered in the unbiased maps. (C) Final refined $2mF_O\text{-}DF_C$ density for the peptide is shown in grey. For β'1-304His6, the experimental map from SHARP following density modification with SOLOMON is shown in blue for the WD-repeat domain (D) and for the 6xHis binding (E). (F) His6 tag final refined $2mF_O\text{-}DF_C$ density from β'1-304His6 shown in grey. (G) View of top surface of β'1-304His6 domain. The His6 tag (purple) from a neighbouring molecule occupies the putative binding site on top of the WD-repeat domain. The CTFKTKTN peptide from the β'1-304 structure in yellow is overlaid with the His6 tag for comparison. The carboxy terminus and -3 residue of the motif (either Lys or His) occupy the same positions in both structures; H306 in the His6 tag sits in the patch formed by D98 and D117, while this patch is occupied by K261 in the CTFKTKTN structure (see Figure 1C).

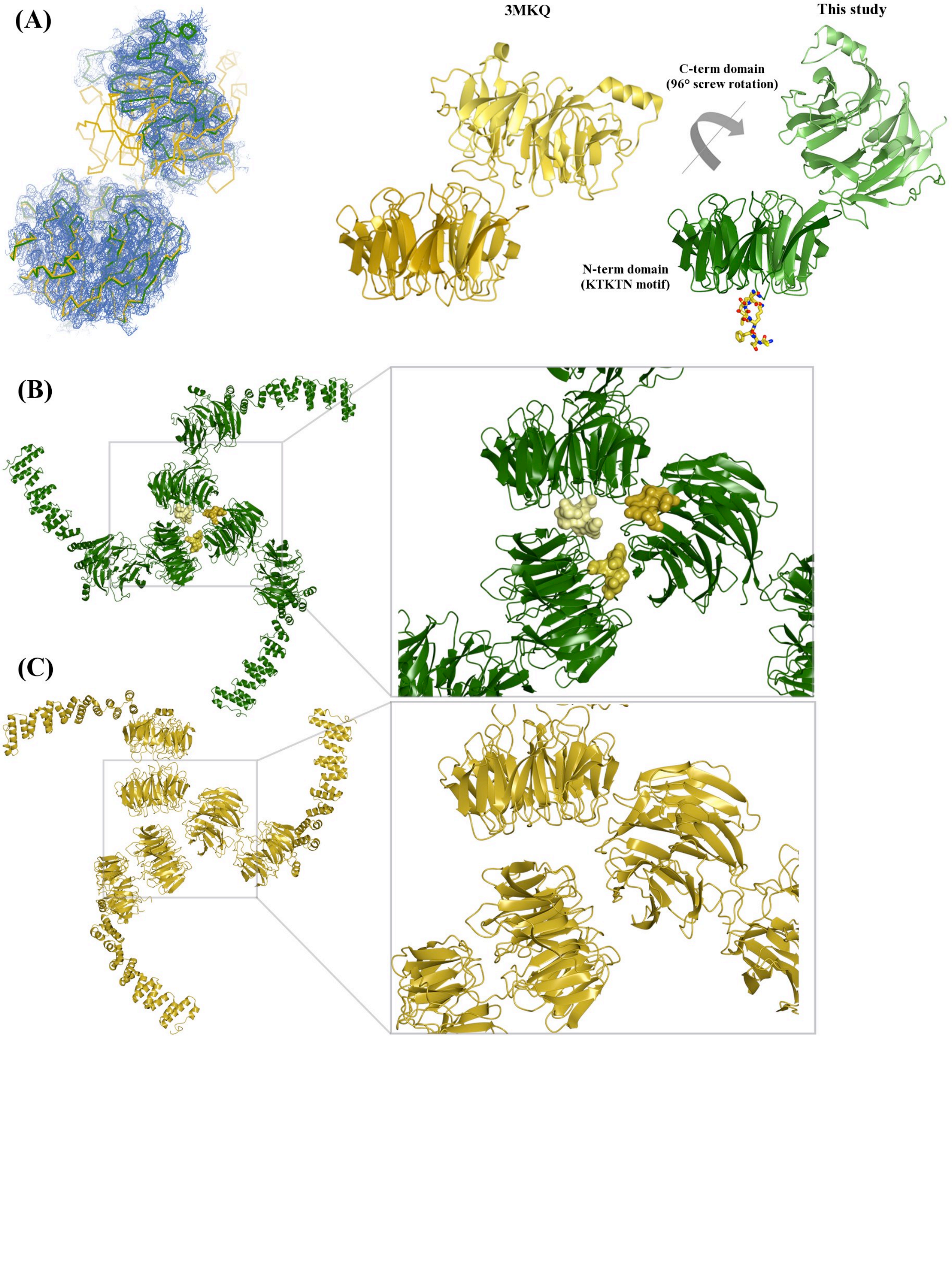
(A)
3MKQ
This study
C-term domain
(96° screw rotation)
N-term domain
(KTKTN motif)
(B)
(C)

Figure S2, related to Figure 1. Flexibility between β'-COP WD-repeat domains facilitates formation of COPI coats having a range of geometries. (A) Electron density following molecular replacement in Phaser indicates that the C-terminal domain (residues 310-604) in our structure (final C-α model in green with peptide omitted) has undergone a relative rotation, when the structures are superimposed on the N-terminal domain 3MKQ (C-α model in gold). Ribbon diagrams of 3MKQ and our structure are shown to right for comparison. (B) Model of trimer based on the conformational change observed in our structure of β'1-604 with CTFKTKTN. There is sufficient space within the proposed trimeric interface for a single KxKxx motif per N-terminal domain. (C) Model of trimer from 3MKQ with trimeric interface shown in the same orientation for comparison. The α-solenoid domains are located ~180° apart in the two models, implying trimers of opposite handedness and further demonstrating the large conformational variability possible in the COPI coat.

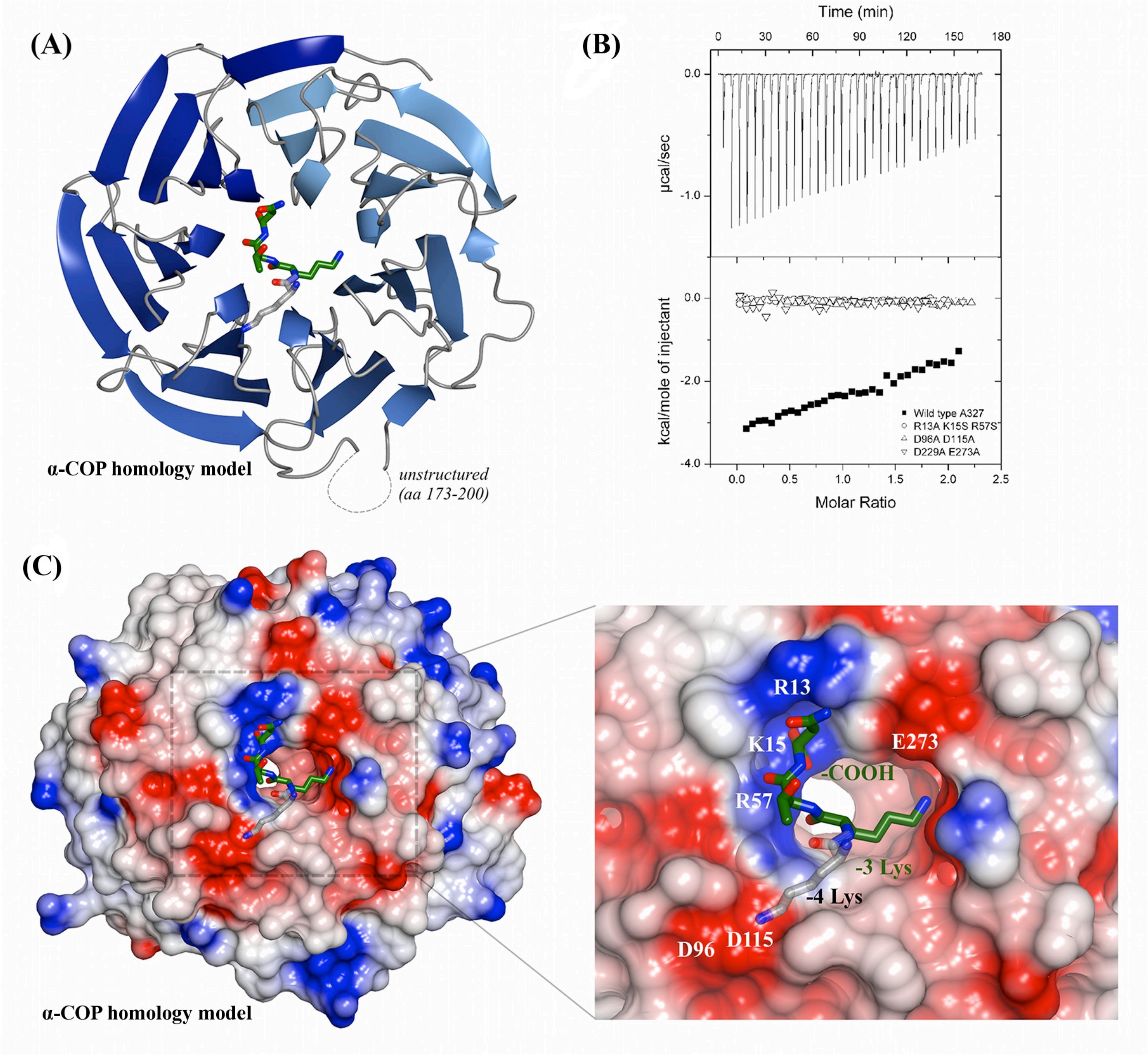

Figure S3, related to Figure 2. Model and *in vitro* data of α-COP 1-327 binding to a KKxx motif. A homology model of residues 1-327 of yeast α-COP binding to the KKTN motif from Wbp1 is shown in ribbon diagram (A) and electrostatic surface potential (C) views. Like β'-COP, α-COP was predicted to form a seven-bladed β-propeller, with residues 173-201 most likely forming an unstructured loop (omitted here). (B) *In vitro* binding studies of α1-327 wild-type and mutants with KKTN motifs. In high salt buffer required for stability (300 mM NaCl), α1-327 bound KKTN with a K_D measured to be 81 ± 9 µM, while point mutants exhibited no measurable binding. (C) The positive patch formed by R13, K15, and R57 binds the carboxy

terminus; the patch formed by D229 and E273 binds the -3 lysine; and a patch formed by D96 and D115 is the most likely candidate for binding the -4 lysine.

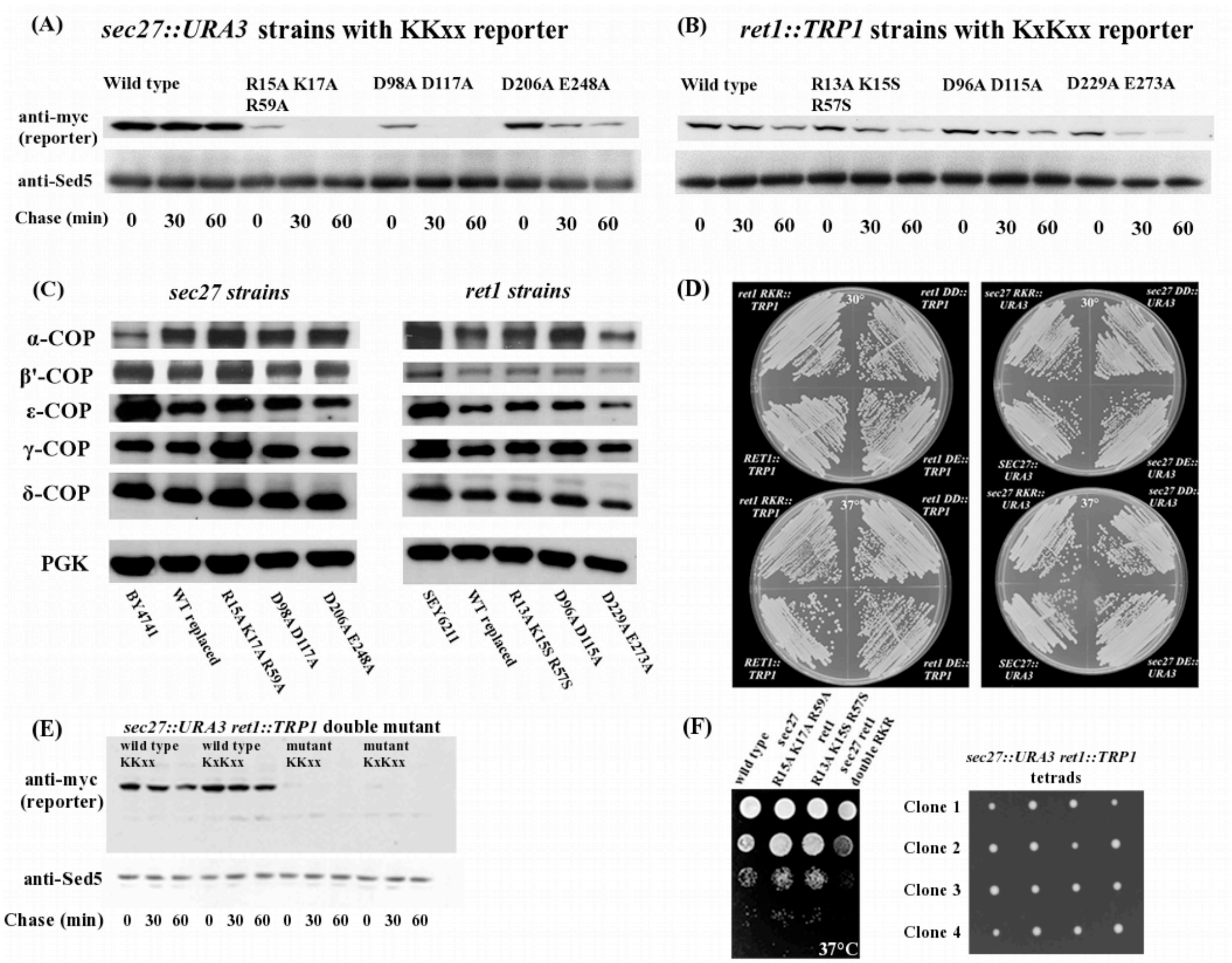

Figure S4, related to Figure 3. Trafficking of converse dilysine reporter constructs, COPI stability, and yeast viability. (A) In *sec27::URA3* (β'-COP) point mutant strains, KKxx reporter constructs exhibit lower steady state levels and are degraded over time. (B) In *ret1::TRP1* (α-COP) point mutant strains, KxKxx reporter constructs exhibit a less dramatic phenotype. Taken together with trafficking of preferred motif-bearing constructs, these data suggest that β'-COP is trafficking the majority of dilysine motif reporter constructs in cells. (C) COPI subunits in both the α/β'/ε and β/δ/γ/ζ subcomplexes are expressed at similar levels in parental, wild-type gene-replaced, and point mutant gene-replaced strains. (D) All single mutant recombinant yeast strains are viable and exhibit no temperature sensitivity after growth on YEPD plates for 48 hours. (E) Trafficking of dilysine-based reporter constructs is severely inhibited in a *sec27::URA3* R15A K17A R59A *ret1::TRP1* R13A K15S R57S double mutant strain lacking carboxy-terminus binding sites for the motif. (F) Tetrads from crossing *sec27::URA3* R15A K17A R59A and

ret1::TRP1 R13A K15S R57S single mutants to generate a double mutant strain are viable. As compared with single mutant strains, the double mutant possesses a slight grown phenotype, as shown in a dilution series.

Table S1. Peptides used in crystallization trials with β'-COP constructs

Peptide	Source	Result
CTFKKTN	Cysteine plus yeast Wbp1 C-terminus	Structures: β'1-304His6, β'1-304
RQEIIKTKLL	Yeast Emp47 C-terminus	No crystals
CTFKTKTN	Synthetic, based on yeast Wbp1	Structures: β'1-304, β'1-604
CTFSGSKTKLL	Synthetic, based on yeast Emp47	Structure: β'1-304, peptide proteolyzed
CTFSGSKTKTN	Synthetic, based on crystal structure	No crystals with K261S
CTSGSKTKTN	Synthetic, based on crystal structure	Crystalline precipitate with K261S
CTGSGSKTKTN	Synthetic, based on crystal structure	Micro-needles only with K261S
SKKYL	Synthetic, yeast two hybrid results	No crystals
HKSKTH	Human UDP-glucuronosyltransferase	No crystals

Table S2, MALS results

Construct	Theoretical mass	Measured mass (2 mg/mL sample)	Measured mass (8 mg/mL sample)
β'1-304	35.5 kDa	34.9 kDa	35.7 kDa
β'1-604	68.8 kDa	70.4 kDa	76.0 kDa

Supplemental Experimental Procedures

Cloning and mutagenesis

β'1-304His6 was placed into a single Nde I site of in-house vector pMW172 under control of a T7 promoter. All GST-tagged constructs (β'1-304GST, β'1-604GST, and α1-327GST) were ligated into Nde I and either HinD III or BamH I sites of pMWGST, a modified form of pMW172 incorporating a C-terminal, thrombin cleavable GST tag. All affinity tags were incorporated at the C-terminal end of the α- or β'-COP WD-repeat domains in order to ensure proper folding.

Site-directed mutagenesis, using wild-type β'1-304GST and α1-327GST constructs as templates, was performed by overlap PCR using complementary primers with the relevant mutation(s) across the site of interest. The final PCR product was digested and placed in the Nde I and BamH I sites of pMWGST. All constructs were sequenced by Beckman Coulter Genomics prior to use.

Protein expression and purification

All constructs expressed at high levels in BL21(DE3)pLysS cells (Invitrogen). Ten milliliter starter cultures were grown from single colonies for six hours at 37°C and used to inoculate one liter flasks of pre-warmed 2xTY media. Cultures were grown at 37°C to an OD_{600} between 0.8-1.0 and expressed at 22°C overnight following induction with 0.2 mM IPTG. All β'-COP constructs were resuspended in 20 mM Tris pH 7.4, 200 mM NaCl, and 2 mM DTT. α-COP constructs required high salt for solubility and were resuspended in 20 mM Tris pH 7.4, 500 mM NaCl, and 2 mM DTT. AEBSF (Calbiochem) was incorporated at early stages of all purifications, and cells were lysed using a disruptor (Constant Systems Ltd).

6xHis-tagged proteins were incubated for one hour at 4°C with 50% Ni-NTA agarose (Qiagen) in buffer, while GST-tagged proteins were incubated with 50% glutathione sepharose (GE Healthcare) in buffer. Protein bound to either matrix was washed extensively with buffer (500 mls to 1 L). For 6xHis-tagged proteins, the wash buffer included 20 mM imidazole to remove contaminants that bind non-specifically to Ni-NTA. 6xHis-tagged proteins were eluted by batch in buffer containing 300 mM imidazole. GST-tagged proteins were cleaved overnight with thrombin (SERVA) at room temperature and eluted by batch in buffer. Following elution, all proteins were concentrated for gel filtration on a S200 preparative column (GE Healthcare).

All α-COP and β'-COP mutants were determined to be folded as judged by circular dichroism and by identical gel fitration elution profiles as compared to wild-type protein (data not shown).

Extended structure determination procedures

General data collection, data processing, and structure determination methods

Data were integrated using iMosflm (Battye et al., 2011) or Xia2 (Winter, 2009), then processed further using either the CCP4 (The CCP4 Suite: Programs for Protein Crystallography., 1994) or PHENIX (Adams et al., 2010) suites. autoSHARP (Vonrheim et al., 2007) was used to locate heavy atom sites and SHARP (Bricogne et al., 2003) was used for experimental phasing of the β'1-304His6 mercury derivative. Phaser (McCoy et al., 2007) was employed for all molecular replacement work. REFMAC5 (Murshudov et al., 1997) was helpful in initial refinement of twinned β'1-604 data. All final refinement runs took place using phenix.refine (Afonine et al., 2005), and *R* factors are reported from phenix.refine. Coot (Emsley and Cowtan, 2004) was used for model building, and final model validation was undertaken in the PHENIX suite using MolProbity (Davis et al., 2007). Structure figures were generated using CCP4MG (Potterton et al., 2002).

Crystallization and structure determination of β'1-304His6

Initially, the most promising construct for structural studies was a C-terminally 6x histidine (His6)-tagged version of β'1-304. Crystallization trials were set up with protein alone as well as in the presence of either the RQEIIKTKLL motif from yeast Emp47 or the CTFKKTN motif, which contained a cysteine residue (for coupling of fluorophores) followed by the final six residues of yeast Wbp1. The best crystals grew only in the presence of the CTFKKTN motif from Wbp1 and were obtained with 10-13 mg/mL of protein and 3 mg/mL peptide by vapor diffusion in 12% PEG1500 (Hampton). Crystals were cryo-protected using 25% glycerol. Mercury derivatives were produced by soaking native crystals in cryo buffer containing 0.5 mM EMTS and soaking for ten minutes prior to flash cooling. Data were collected at Diamond Light Source, beamline I02, from crystals flash frozen by plunging into liquid nitrogen.

Native crystals diffracted beyond 1.8 Å and were of space group C2 with unit cell dimensions a=171.5 Å b=50.5 Å c=74.5 Å α=90° β=103.99° γ=90°. The structure was

determined using single isomorphous replacement with anomalous scattering (SIRAS); two mercury sites were identified by autoSHARP. Phasing was subsequently undertaken in SHARP, together with density modification with SOLOMON (Abrahams and Leslie, 1996). Solvent content was optimized at 47.5% with two copies in the asymmetric unit. Buccaneer (Cowtan, 2006) was used for initial rounds of automated model building, with final building in Coot. The structure was refined to final R/R_{free} values of 0.1880/0.2233. Density for all 310 residues was clear and unambiguous. Representative samples of density are shown in Figure S1D-F.

Attempts to displace the 6xHis binding in the crystal were made by soaking in a very high concentrations of the KTKLL motif from yeast Emp47. Inspection of the resulting density indicated that the His-tag was at least partially displaced, and to some degree, we could observe the side chains of the final three residues in the motif, including the C-terminal leucine (data not shown). However, the density was certainly ambiguous, probably as a result of the 6xHis tag and peptide competing for the same binding site in this particular crystal form, and this line of experimentation was not pursued further.

For all subsequent crystal trials, the β'1-304GST construct was used following cleavage of GST. A number of peptides (Table S1) were used in co-crystallization trials.

Structure determination of β'1-604 with CTFKTKTN

β'1-604 crystallized in the presence of CTFKTKTN in 0.1M bicine pH 9.0, 10% MPD. The crystals were cryo-protected using 40% MPD, flash frozen by plunging in liquid nitrogen, and data were collected to 3.0 Å at Diamond Light Source on beamline I04-1, using a Pilatus 2M detector. The structure was solved by molecular replacement in Phaser using two successive searches. The high resolution β'1-304His6 structure was used first as a model to locate the N-terminal WD-repeat domain, and in a second search, residues 310-600 of PDB ID: 3MKQ were used to locate the C-terminal domain. Using residues 1-604 of 3MKQ alone as a search model did not yield the correct solution because of the screw rotation of the C-terminal WD-repeat domain; movement of this domain is clear in the $2mF_O\text{-}DF_C$ electron density following molecular replacement in Phaser (Figure S2A). The crystals were of space group $P3_1$ with cell dimensions a=127.25 Å b=127.25 Å c=59.06 Å α=90° β=90° γ=120° and exhibited twinning (two domains of fractions 58.6 and 41.4%). Intensity-based twin refinement in REFMAC5 was used for initial rounds of refinement. The final structure was refined to R/R_{free} of 0.2396/0.2943

in phenix.refine. All eight residues of the peptide and residues 1-601 of β'-COP are visible, with a single break in main chain density at amino acids 338-339.

Homology model of the α-COP N-terminal propeller

Despite extensive efforts, all attempts to crystallize the human, yeast, or *Drosophila* α-COP N-terminal WD-repeat domain, either on its own or in the presence of multiple dilysine-based peptides, failed. As compared to β'-COP, α-COP was ill-behaved and required high salt (500 mM NaCl) to maintain solubility. Attempts to engineer the WD-repeat domain for crystallization by methylation or by removing the predicted flexible loop (residues 173-200) to facilitate crystal packing also failed. We have instead produced a homology model of the yeast α-COP N-terminal WD-repeat domain (residues 1-327; Figure S2) with MODELLER (Sali et al., 1995), using our β'1-304/KxKxx motif structure as a template. Residues 173-200 have been omitted for clarity, as they are predicted to form an unstructured surface loop between blades four and five of the seven-bladed propeller.

Sequence alignments

In order to map residue conservation onto the structure of β'-COP 1-304, sequences of the N-terminal WD-repeat domains of both α- and β'-COP from *S. cerevisiae*, *C. elegans*, *D. melanogaster*, *A. thaliana, G. gallus*, *M. musculus*, and *H. sapiens* were aligned using ClustalW (Larkin et al., 2007).

Isothermal titration calorimetry

ITC experiments were conducted on a Microcal VP-ITC machine (GE Healthcare) at 10°C, using 35 injections of 8 μL each with a 300 sec interval between injections. The molar peptide concentration in the syringe was ten times that of the protein in the cell. Titration data were analyzed using ORIGIN software in order to obtain values for stoichiometry (N), equilibrium association constant (K_a), and enthalpy of binding. All experiments were carried out a minimum of three times and appropriate standard deviations are reported.

Wild type and mutant constructs containing a C-terminal GST tag were used to prepare proteins for ITC experiments; all GST tags were removed using thrombin as described above prior to ITC runs. All peptides were resuspended in exactly the same batch of buffer as protein to

ensure minimal dilution effects. β'-COP constructs were gel filtered into 50 mM Hepes pH 7.4, 100 mM NaCl to mimic physiological salt conditions. Wild type yeast α-COP (α1-327) was gel filtered into 20 mM Hepes pH 7.4, 500 mM NaCl. Immediately prior to a run, the protein was desalted over a PD-10 column (GE Healthcare) into 20 mM Hepes pH 7.4, 300 mM NaCl, the minimum ionic strength at which α-COP remains soluble during the course of a standard run.

Control runs of β'-COP with KxKxx were performed in 300 mM NaCl and compared to K_D values obtained in 100 mM NaCl in order to estimate a true K_D for wild type α-COP binding to KKTN because a charge-mediated interaction in higher salt will give artificially weak measurements. The measured K_D of β'-COP with KTKLL is four- to five-fold weaker at 300 mM NaCl than at 100 mM NaCl. Thus, we estimate the K_D for α-COP binding to KKTN to be closer to 10-20 μM, which is the approximate K_D for β'-COP binding to the KTKLL motif. As a result of the high salt concentrations required, we cannot detect binding of α-COP to KxKxx motifs.

Multi-angle light scattering (MALS) experiments with β'1-304 and β'1-604

Multi-angle light scattering (MALS) experiments were performed immediately following size-exclusion chromatography by inline measurement of static light-scattering (DAWN HELEOS II, Wyatt Technology), differential refractive index (Optilab rEX, Wyatt Technology) and ultraviolet absorbance (Agilent 1200 UV, Agilent Technologies). Samples of β'1-304 or 1-604 (100 μL) were injected onto an analytical S200 10/300 gel filtration column (GE Healthcare) equilibrated in 50 mM Hepes pH 7.4 and 100 mM NaCl at a flow rate of 0.4 mL/min. Data were analyzed using the ASTRA V (Wyatt Technology) with a dn/dc value set to 0.186 mL/g. Results are summarized in Table S2, measured at two different protein concentrations. At higher concentrations, β'1-604 is starting to self-associate, most likely at the C-terminal end, because the construct lacks the α-solenoid domain.

Generation of yeast strains by homologous recombination

Wild-type *SEC27* (β'-COP) and *RET1* (α-COP) genes were cloned into Not I/Sal I sites of yeast vectors pRS314 and 414, respectively, with endogenous promoter and terminator sequences, as well as the intron for *SEC27*. A selectable marker (*URA* for *SEC27* and *TRP* for

RET1) was placed in each terminator region after first introducing a unique Nde I site, and these strains were used as templates for site-directed mutagenesis. Genes of interest were amplified by PCR to produce linear fragments for transforming the parental yeast strain BY4741 for *SEC27* strains and SEY6211 for *RET1* strains. Colonies were screened for the selectable marker and then sequenced to verify incorporation of mutations. As controls, wild-type replacement versions (*SEC27::URA3*, *RET1::TRP1*) were generated. These strains were then used as templates for making mutants, which are summarized below. Relevant mutations were incorporated by site-directed mutagenesis using a fragment formed by a unique Not I and internal EcoR I site.

List of recombinant yeast strains

Name	Point mutations	Description
BY4741	None	MATa *uraΔ0 leu2Δ0 his3Δ1 met15Δ0* (parental)
SEC27::URA3	None	Wild type replacement control with *URA3* cassette
sec27 RKR::URA3	R15A K17A R59A	Disrupts binding of reporter C-terminus to β'-COP
sec27 DD::URA3	D98A D117A	Disrupts binding of reporter -5 lysine to β'-COP
sec27 DE:: URA3	D206A E248A	Disrupts binding of reporter -3 lysine to β'-COP
SEY6211	None	MATa *ura3-52 leu2-3, -112 his3-Δ200 trp1-Δ901 ade2-101 suc2-Δ9*
RET1::TRP1	None	Wild type replacement control with *TRP1* cassette
ret1 RKR::TRP1	R13A K15S R57S	Disrupts binding of reporter C-terminus to α-COP
ret1 DD::TRP1	D96A D115A	Disrupts binding of reporter -4 lysine to α-COP
ret1 DE::TRP1	D229A E273A	Disrupts binding of reporter -3 lysine to α-COP

Yeast Emp47 under control of its endogenous promoter was used as the backbone for reporter constructs, and a myc-tag was incorporated following the transmembrane domain to facilitate Western blotting. The tagged reporter plasmid used was derived from that described by (Schröder et al., 1995), and subsequent mutations were made by PCR, using a SpeI site inserted at the end of the ORF and mutations were verified by sequencing. The reporter was carried on pRS415. The KxKxx reporter contained the endogenous Emp47 transmembrane domain and cytoplasmic sequence (RQEIIKTKLL). The KKxx reporter contained the transmembrane domain of Emp47 followed by the cytoplasmic domain of Wbp1 (LETFKKTN).

The double mutant strain *sec27::URA3 ret1::TRP1* was generated by first creating the *ret1* R15A K17S R57S mutant in alpha mating type strain SEY6211 and verified by PCR and sequencing. The strain was mated with the original *sec27::URA3* R15A K17A R59A strain and

sporulated. Tetrads were picked using a Singer micro-dissection apparatus and plated onto YEPD. Tetrads were analyzed for auxotrophic markers, followed by PCR screening and sequencing to confirm the genotype. Growth phenotypes were tested at 37°C on YEPD overnight.

Trafficking of dilysine-based reporter constructs in yeast

The relevant dilysine reporter construct was next introduced into appropriate strains. The KxKxx reporter construct was introduced into *SEC27* wild type and mutant replaced strains, while the KKxx reporter was introduced into *RET1* wild type replaced and mutant strains. Reporter levels were then monitored by Western blotting against the myc tag both prior to and following a 20 μg/mL cycloheximide chase at time points of 0, 30, and 60 minutes. Protein extracts were prepared according to the alkaline lysis method (Volland et al., 1994). Western blots were probed with monoclonal anti-myc (Sigma) and rabbit anti-Sed5 serum (Hardwick and Pelham, 1992). Graphical representations of the data were made using ImageJ software (Abramoff et al., 2004).

Antibodies

The following antibodies were used for immunoblotting in parental, wild type replaced, and mutant yeast strains: anti-Ret1p (Letourneur et al., 1994) for α-COP, anti-Sec27p (Duden et al., 1994) for β'-COP, anti-Ret2p (Cosson et al., 1996) for δ-COP, anti-Sec21p (Hosobuchi et al., 1992) for γ-COP, and anti-Sec28p (Duden et al., 1998) for ε-COP. Anti-PGK (Molecular Probes) was used as a loading control, and anti-Sed5p (Eugster et al., 2004) was used as a marker for retrograde trafficking. Lysates were prepared from yeast cells in log phase growth using alkaline lysis and TCA precipitation (Volland et al., 1994).

Supplemental References

Abrahams, J. P., and Leslie, A. G. (1996). Methods used in the structure determination of bovine mitochondrial F1 ATPase. Acta Cryst D *52*, 30–42.

Abramoff, M. D., Magalhaes, P. J., and Ram, S. J. (2004). Image processing with ImageJ. Biophotics Int *11*, 36–42.

Adams, P. D., Afonine, P. V., Bunkóczi, G., Chen, V. B., Davis, I. W., Echools, N., Headd, J. J., Hung, L.-W., Kapral, G. J., Grosse-Kunstleve, R. W., et al. (2010). PHENIX: a comprehensive, Python-based system for macromolecular structure solution. Acta Cryst D *66*, 213–221.

Afonine, P., Grosse-Kunstleve, R. W., and Adams, P. D. (2005). phenix.refine. CCP4 Newsletter *42*, Contribution 8.

Battye, T. G. G., Kontogiannis, L., Johnson, O., Powell, H. R., and Leslie, A. G. (2011). iMOSFLM: a new graphical interface for diffraction-image processing with MOSFLM. Acta Cryst D *67*, 271–281.

Bricogne, G., Vonrhein, C., Flensburg, C., Schiltz, M., and Paciorek, W. (2003). Generation, representation and flow of phase information in structure determination: recent developments in and around SHARP 2.0. Acta Cryst D *59*, 2023–2030.

Cosson, P., Demolliere, C., Hennecke, S., Duden, R., and Letourneur, F. (1996). δ- and ζ-COP, two coatomer subunits homologous to clathrin-associated proteins, are involved in ER retrieval. EMBO J *15*, 1792–1798.

Cowtan, K. (2006). The Buccaneer software for automated model building. Acta Cryst D *62*, 1002–1011.

Davis, I. W., Leaver-Fay, A., Chen, V. B., Block, J. N., Kapral, G. J., Wang, X., Murray, L. W., Arendall 3rd, W. B., Snoeyink, J., Richardson, J. S., et al. (2007). MolProbity: all-atom contacts and structure validation for proteins and nucleic acids. Nucleic Acids Res *35*, W375–83.

Duden, R., Hosobuchi, M., Hamamoto, S., Winey, M., Byers, B., and Schekman, R. (1994). Yeast β and β'-Coat Proteins (COP): Two coatomer subunits essential for endoplasmic reticulum-to-Golgi protein traffic. Yeast *269*, 24486–24495.

Duden, R., Kajikawa, L., Wuestehube, L., and Schekman, R. (1998). ε-COP is a structural component of coatomer that functions to stabilize α-COP. EMBO J *17*, 985–995.

Emsley, P., and Cowtan, K. (2004). Coot: model-building tools for molecular graphics. Acta Cryst D *60*, 2126–2132.

Eugster, A., Frigerio, G., Dale, M., and Duden, R. (2004). The α- and β'-COP WD40 Domains Mediate Cargo-selective Interactions with Distinct Di-lysine Motifs. Mol Biol Cell *15*, 1011–1023.

Hardwick, K. G., and Pelham, H. R. (1992). SED5 encodes a 39-kD integral membrane protein required for vesicular transport between the ER and the Golgi complex. J Cell Biol *119*, 513–521.

Hosobuchi, M., Kreis, T., and Schekman, R. (1992). SEC21 is a gene required for ER to Golgi protein transport that encodes a subunit of yeast coatomer. Nature *360*, 603–605.

Larkin, M., Blackshields, G., Brown, N., Chenna, R., McGettigan, P., McWilliam, H., Valentin, F., Wallace, I., Wilm, A., Lopez, R., et al. (2007). ClustalW and ClustalX version2. Bioinformatics *23*, 2947–2948.

Letourneur, F., Gaynor, E. C., Hennecke, S., Démollière, C., Duden, R., Emr, S. D., Riezman, H., and Cosson, P. (1994). Coatomer is essential for retrieval of dilysine-tagged proteins to the endoplasmic reticulum. Cell *79*, 1199–1207.

McCoy, A. J., Grosse-Kunstleve, R. W., Adams, P. D., Winn, M. D., Storoni, L. C., and Read, R. J. (2007). Phaser Crystallographic Software. J Appl Cryst *40*, 658–674.

Murshudov, G. N., Vagin, A. A., and Dodson, E. J. (1997). Refinement of macromolecular structures by the maximum-likelihood method. Acta Cryst D *53*, 240–255.

Potterton, E., McNicholas, S., Krissinel, E., Cowtan, K., and Noble, M. (2002). The CCP4 molecular graphics project. Acta Cryst D *58*, 1955–1957.

Sali, A., Potterton, L., Yuan, F., van Vlijmen, H., and Karplus, M. (1995). Evaluation of comparative protein modeling by MODELLER. Proteins *23*, 318–326.

Schröder, S., Schimmöller, F., Singer-Krüger, B., and Riezman, H. (1995). The Golgi-localization of yeast Emp47p depends on its di-lysine motif but is not affected by the ret1-1 mutation in α-COP. J Cell Biol *131*, 895–912.

The CCP4 Suite: Programs for Protein Crystallography. (1994). Acta Cryst D *50*, 760–763.

Volland, C., Urban-Grimal, D., Géraud, G., and Haguenauer-Tsapis, R. (1994). Endocytosis and degradation of the yeast uracil permease under adverse conditions. J Biol Chem *269*, 9833–9841.

Vonrheim, C., Blanc, E., Roversi, P., and Bricogne, G. (2007). Automated structure solution with autoSHARP. Methods Mol Biol *264*, 215–230.

Winter, G. (2009). Xia2: an Expert System for Macromolecular Crystallography Data Reduction. J Appl Crystallog *43*, 186–190.
